# Untangling Irregular Actin Cytoskeleton Architectures in Tomograms of the Cell with *Struwwel Tracer*

**DOI:** 10.3390/ijms242417183

**Published:** 2023-12-06

**Authors:** Salim Sazzed, Peter Scheible, Jing He, Willy Wriggers

**Affiliations:** 1 Department of Computer Science, Old Dominion University, Norfolk, VA 23529, USA; ssazz001@odu.edu (S.S.);; 2 Department of Mechanical and Aerospace Engineering, Old Dominion University, Norfolk, VA 23529, USA

**Keywords:** cryo-electron tomography, segmentation, filament tracing

## Abstract

In this work, we established, validated, and optimized a novel computational framework for tracing arbitrarily oriented actin filaments in cryo-electron tomography maps. Our approach was designed for highly complex intracellular architectures in which a long-range cytoskeleton network extends throughout the cell bodies and protrusions. The irregular organization of the actin network, as well as cryo-electron-tomography-specific noise, missing wedge artifacts, and map dimensions call for a specialized implementation that is both robust and efficient. Our proposed solution, *Struwwel Tracer*, accumulates densities along paths of a specific length in various directions, starting from locally determined seed points. The highest-density paths originating from the seed points form short linear candidate filament segments, which are further scrutinized and classified by users via inspection of a novel *pruning map*, which visualizes the likelihood of being a part of longer filaments. The pruned linear candidate filament segments are then iteratively fused into continuous, longer, and curved filaments based on their relative orientations, gap spacings, and extendibility. When applied to the simulated phantom tomograms of a *Dictyostelium discoideum* filopodium under experimental conditions, *Struwwel Tracer* demonstrated high efficacy, with F1-scores ranging from 0.85 to 0.90, depending on the noise level. Furthermore, when applied to a previously untraced experimental tomogram of mouse fibroblast lamellipodia, the filaments predicted by *Struwwel Tracer* exhibited a good visual agreement with the experimental map. The *Struwwel Tracer* framework is highly time efficient and can complete the tracing process in just a few minutes. The source code is publicly available with version 3.2 of the free and open-source *Situs* software package.

## 1. Introduction

Shape, motility, and transport within a eukaryotic cell are based on an extensive actin cytoskeleton [1]. To visualize actin filaments in their native state, researchers commonly employ cryo-electron tomography (cryo-ET), a specialized imaging technique that enables 3D insight into the internal cellular structure. Cryo-ET has recently shown that actin filaments provide a “missing evolutionary link” between Archaea and complex eukaryotic life forms, such as animals and plants [2]. The cryo-ET by Pilhofer’s lab at ETH Zurich supports an emerging hypothesis that extensive cytoskeletal actin structures arose first in the Asgard Archaea, before the appearance of the first eukaryotes on Earth, and could, therefore, have contributed to the emergence of complex organisms [3]. Moreover, the observed cytoskeletal protrusions in the Archaea suggest a detailed mechanism for eukaryogenesis, in which a primordial Asgard archaeon (the closest known relative of eukaryotes) interacts with the predecessor of the bacterial endosymbiont by means of the actin-powered protrusions and eventually endogenizes it [2,3].

Cryo-ET involves capturing a series of 2D images of a specimen at extremely low temperatures, preserving its frozen state while maintaining its hydrated environment. This enables detailed imaging of actin filaments without disrupting their native structure. These 2D projections are subsequently aligned using various computational techniques, collectively referred to as 3D reconstruction, to generate the 3D tomogram. The 3–5 nm-resolution tomograms obtained by cryo-ET typically present considerable noise, induced by the limited electron dose used in the image acquisition, and they exhibit directional artifacts from the absence of certain view directions (“missing wedge” in the Fourier space [4]), caused by the limited tilt range of the specimen holder in the microscope.

Determining the organization of actin filaments is highly important as they are responsible for a variety of cellular processes, such as muscle contraction, transport, and cell motility [1], and therefore, it can aid in characterizing experimental, dynamic, or pathological changes in the cytoskeleton [5]. Actin filaments are thin and flexible. They assemble to form diverse organizational patterns, from hexagonal closely packed bundles in the shaft of hair cell stereocilia [6], to directionally biased semi-ordered strands in the stereocilium taper region [4,7], to irregular, randomly oriented actin networks in filopodia [8]. The three types of actin organization can be likened to the appearance of a person’s hair in various stages of tidiness (brushed, windblown, unkempt), and each type requires specialized algorithms for their computational interpretation. Our recent algorithm development was mainly focused on ordered or semi-ordered patterns in stereocilia, where filaments form strands with a mean direction that can be exploited in directional denoising or deconvolution [4,6,7]. In many cells, however, such as in the ancestral Archaea [2], the filaments are randomly oriented and form highly irregular networks, which defy any directionally biased denoising approaches. In this work, we present a novel approach, *Struwwel Tracer*, to quantitatively organize such important disorganized actin networks. (The tool was named after Heinrich Hoffmann’s famously shaggy Struwwelpeter character.)

The presence of noise, artifacts, and other structures, such as actin-binding proteins, can obfuscate the filaments and make the tracing difficult. Distinguishing true filaments from noise or other structures in tomograms requires careful examination and expertise, which ultimately make manual tracing of actin filaments a labor-intensive task that requires significant time and effort [8,9,10]. In addition, manual tracing is often subjective in nature and relies heavily on the annotator’s interpretation. The reliability and reproducibility of the annotation procedure are greatly compromised by factors such as low contrast, various types of artifacts, and inherent uncertainty. This is particularly evident when the procedure is applied directly to whole-cell tomograms (i.e., without any subtomogram averaging) with typical resolutions below 2 nm and varying in orientation due to the missing wedge effect. Moreover, when dealing with large datasets containing 2D image stacks, the repetitive nature of the annotation task increases the chance of error, such as an inadvertent skipping between neighboring filaments. To mitigate these potential errors and to make the tracing objective and reproducible, it becomes necessary to develop automated approaches that can be independently validated.

Over the years, a number of automatic methods have been developed to trace actin filaments [4,8,10,11,12,13]; however, the existing methods have certain limitations:

1. A significant portion of these approaches can be ruled out for the present work because they are only applicable to non-actin filaments such as microtubules [14] and polysaccharides [15], or to actin filaments observed in relatively clean light or confocal microscopy images [16,17].
2. Among the cryo-ET-related actin tracing methods, some are only applicable for the tracing of well-ordered filaments [4,6,7]. Among these, one noteworthy tool is our *Spaghetti Tracer* approach [7]. *Spaghetti Tracer* introduced a paradigm shift in the tracing of semi-regular actin filaments because it is a dynamic-programming-based method at the voxel level that does not require an expensive missing wedge correction, template convolution, or deconvolution. Therefore, it yields a substantial improvement in time efficiency over template convolution [8,10] or deconvolution methods [4], enabling fast and accurate tracing of such filament arrangements. (The accuracy of *Spaghetti Tracer* was validated in a rigorous statistical analysis, achieving F1-scores of 0.86–0.95 on phantom tomograms under experimental conditions.) The success of *Spaghetti Tracer* motivated us to extend its capabilities to randomly oriented actin filaments in the present work.
3. There are very few earlier algorithms that are agnostic of the relative orientations and distances of the actin filaments, so that they can trace central lines of irregular filaments individually without leveraging the information of adjacent filaments or requiring a mean direction. *Volume Tracer* [8] utilized an expensive genetic-algorithm-based search employing a population of cylindrical templates (combined with a bi-directional tracing) to detect randomly oriented filaments in *Dictyostelium discoideum* filopodia. The genetic algorithm was implemented as part of our group’s free, open-source *Situs* and *Sculptor* packages, but it required extensive computational time on the order of several days when applied to a complete tomogram, without guaranteeing convergence (leading to false negatives when a user is impatient). Co-author Rigort also developed a similar template-matching method independently [10]. This approach was implemented in *Amira*, a commercial software requiring a paid license and limiting any algorithmic modifications by third parties or end users.
4. Recently, a number of deep-learning-based approaches have been proposed for the segmentation of diverse biological assemblies, including actin [18,19]. For example, Chen et al. [18] presented a deep-learning-based segmentation approach for a voxel-level classification of shapes of interest in the tomogram and integrated the approach into the *EMAN2* [20] package. However, these segmentation tools are generic in nature and are not specifically designed for filamentous shape structures. Besides, they require users to annotate training data and fine-tune the deep learning model, which could be a laborious process. These segmentation approaches only provide a voxel-level density segmentation, without any tracing of central lines. Recent studies that used these segmentation methods subsequently required separate approaches, such as the above template matching, for the additional tracing [11,12,13].

**Figure 1:**
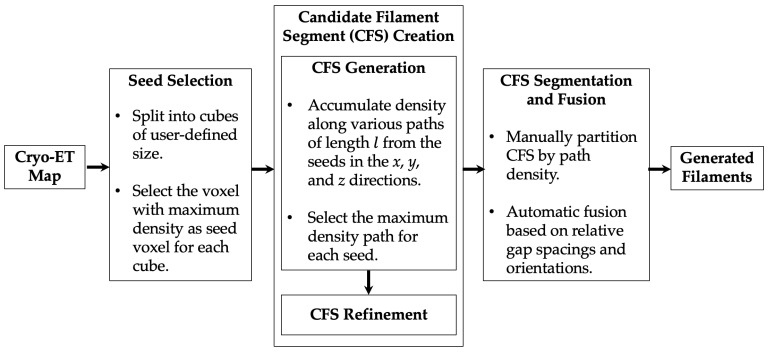
Overall flow diagram of *Struwwel Tracer* as described in Section 3.

In summary, the current state-of-the-art of identifying actin filament networks in cryo-ET has several drawbacks, including the fragmentation of tools and packages, the prerequisite to buy commercial software (*Amira*), complex manual interventions (e.g., manual annotation for segmentation or the training of deep learning networks), and computational expense. Our newly proposed approach, demonstrated in Section 2, can address all these drawbacks. *Struwwel Tracer* is an efficient, accurate, free, open-source tool for tracing randomly oriented filaments in actin networks (Figure 1). We describe in in Section 3 how the framework first detects local seed points (Figure 1) in the map, from which it generates short candidate filament segments (CFSs). Next, path densities are accumulated by exploring all potential filament paths within 45${}^{\circ }$ search pyramids from the *x*, *y*, and *z* directions. After placing CFSs across the map based on the maxima of path densities, we describe in Section 3 how a manual segmentation can be efficiently performed by visualizing an intermediate “pruning map” in a third-party molecular graphics program. Finally, the surviving CFSs are iteratively fused (Figure 1) into longer, curved filaments based on their relative orientations and gap spacings after extension.

## 2. Results and Discussion

To evaluate the performance of *Struwwel Tracer*, a comprehensive statistical F1-score analysis was performed on simulated tomograms of a *Dictyostelium discoideum* filopodium with a known ground truth. Different levels of noise were added to simulate realistic imaging conditions. Additionally, the effectiveness of *Struwwel Tracer* was demonstrated on an experimental tomogram of a mouse fibroblast lamellipodium that was interpreted for the first time by filament tracing. Finally, we report the implementation details and computation times of our software.

### 2.1. Statistical Evaluation of Tracing Accuracy in Simulated Tomograms

In earlier work [7], we established a rigorous statistical evaluation protocol [5] for testing the accuracy of filament-tracing approaches. This protocol is based on simulated phantom tomograms that we generated from known filament traces under realistic conditions that matched the noise profile and missing wedge properties of an experimental tomogram (see Section 3.1). To evaluate *Struwwel Tracer*, we computed the precision, recall, and F1-scores based on the observed agreement.

The ground truth filament voxels of simulated *Dictyostelium discoideum* filopodium maps (see Section 3.1) were compared with the filament voxels predicted by the tracing. Due to the inherent high noise levels present in cryo-ET images, previous studies [5] have highlighted the limitations of conducting one-to-one voxel-level comparisons between the ground truth and predictions. Therefore, here, we used a more-pragmatic approach; rather than direct voxel-level comparison, we performed the comparison based on a neighborhood range [5,7] of three voxels. The true positive (TP), false positive (FP), and false negative (FN) voxels were then computed in the following way:

*True positive:* If a ground truth filament voxel is found within a 3×3×3-voxel neighborhood of a predicted voxel, it is considered a TP.

*False positive:* If no ground truth voxel is found within a 3×3×3-voxel neighborhood of a predicted voxel, it is considered an FP.

*False negative:* If no predicted voxel is found within a 3×3×3-voxel neighborhood of the ground truth voxel, it is considered an FN.

The recall (*R*), precision (*P*), and their harmonic mean (F1) are then computed according to

(1) $$R=\frac{TP}{TP+FN},$$

(2) $$P=\frac{TP}{TP+FP},$$

(3) $$F1=\frac{2*R*P}{R+P}.$$

Table 1 presents the precision, recall, and F1-scores obtained by *Struwwel Tracer* in simulated tomograms with varied levels of noise (see Section 3.1). *Struwwel Tracer* achieved a high F1-score of 0.90 when applied to the lowest-noise map we tested. The high precision score of 0.97 indicated that the framework recognized mostly true filaments in the simulated tomogram (FP predictions were negligible). As the noise level increased, the performance degraded a bit due to the slightly lower recall values (i.e., a small number of true filaments were missed); however, the obtained F1-scores still remained above 0.8. Given the inherent noise in the tomogram, the recall scores ranged slightly below the precision scores since missing filaments’ densities resulted in FNs.

**Figure 2:**
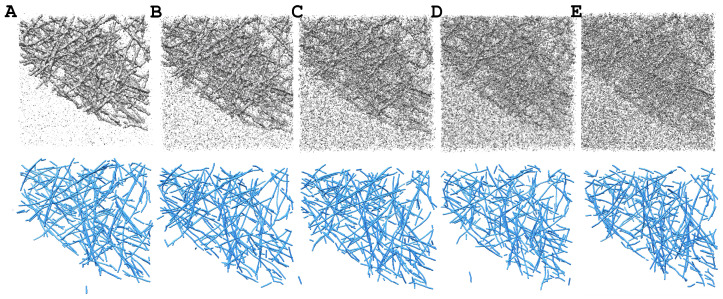
The simulated tomograms (top row) used in this study (isocontour level: mean + 1.5 × standard deviation) and the corresponding *Struwwel Tracer* predictions (bottom row). The simulated tomograms are shown at the following noise levels (see Section 3.1): (**A**) 0.35, (**B**) 0.50, (**C**) 0.65, (**D**) 0.80, and (**E**) 0.95. All molecular graphics in this work were created with UCSF Chimera [22].

Nevertheless, the observed F1-scores ranging from 0.85 to 0.90 (illustrated in Figure 2 for noise levels of 0.35 to 0.95) presented a remarkable level of accuracy for a density-based structure prediction. For comparison, we achieved much lower F1-scores of 0.72 for alpha helices and 0.65 for beta sheets in a recent state-of-the-art deep learning prediction of secondary structure features in cryo-electron microscopy maps [21]. The high *Struwwel Tracer* F1-scores were only slightly below those observed earlier with *Spaghetti Tracer* (0.86–0.95) on much-more-ordered actin filaments [7].

### 2.2. Measuring Filament Center Lines in a Previously Untraced Experimental Tomogram

We applied *Struwwel Tracer* to an experimental tomogram of a mouse fibroblast lamellipodium [23], which was deposited by the authors in the Electron Microscopy Data Bank (EMDB) [24] as EMD-11870. This specific map has not been computationally traced due to their focus on Arp2/3 protein branch junctions in [23], so a measurement of actin center lines could provide complementary information to the paper. (Tracings were shown for similar lamellipodia tomograms in [11], but only EMD-11870 is publicly available).

**Figure 3:**
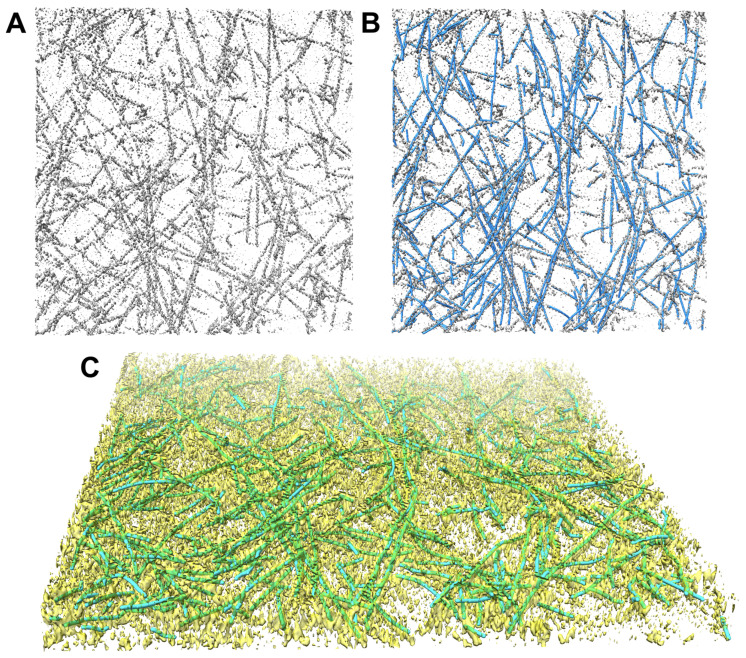
(**A**) A 312 × 320 × 20 subtomogram cropped from EMD-11870 [23] map indices *i* = 100–411, *j* = 200–519, and *k* = 74–93. The isocontour level in the rendering is 0.4518 (mean + 1.5 × standard deviation). (**B**) The map density in (**A**) overlayed by the *Struwwel Tracer*-predicted filament center lines (blue). (**C**) The 3D perspective rendering of the map density in (**A**) (yellow) overlayed by the *Struwwel Tracer*-predicted filament center lines (green).

Since this was a previously untraced map and we did not have any ground truth (i.e., no annotated model), we provide in Figure 3 a visual comparison to demonstrate the excellent agreement between the density and the predicted filament center lines. EMD-11870 [23] is a larger tomogram that contains randomly oriented and branched actin filaments. For our demonstration in Figure 3A, we selected a 312 × 320 × 20 sub-region of the tomogram in which filamentous patterns of actin can be clearly appreciated. The *Struwwel Tracer* prediction results are illustrated in Figure 3B,C.

### 2.3. Computation Time and Manual Intervention

In terms of computational efficiency, *Struwwel Tracer* demonstrated outstanding performance, surpassing the earlier tracing method [8] (which took days of computational time) by several orders of magnitude in speed. On an Apple MacBook Pro equipped with a 2.6-GHz Intel Core i7 processor, we observed that, for a simulated tomogram of size 200 × 200 × 71 voxels, *Struwwel Tracer* took approximately three minutes of runtime. Given the ongoing developments in cryo-ET, it is expected that hundreds of tomograms can soon be acquired within a few days [11]. *Struwwel Tracer* would be able to match this processing speed.

In addition, the proposed method does not require extensive manual intervention, which is the case for interactive and deep-learning-based tools for which users either need to tune many parameters or label training data and train the model for prediction. For the *Struwwel Tracer* approach, user intervention is needed only once in the CFS segmentation step to select an approximate value of the threshold in the pruning map. This requires opening the pruning map in visualization software and takes at most a few minutes.

### 2.4. Software Implementation and Dissemination

The newly developed *Struwwel Tracer* approach is set to be seamlessly integrated into version 3.2 of *Situs*,a widely used, open-source software package for biological image interpretation (see the Data Availability Statement). *Struwwel Tracer* will be disseminated with *Situs* Version 3.2 as a new command line tool named *strwtrc* (following the seven-letter naming convention). To ensure full compatibility with *Situs*, *strwtrc* was implemented using C/C++, the primary language of *Situs*. A notable advantage of *strwtrc* is that it does not rely on any third-party libraries, making it self-contained and efficient. Moreover, *strwtrc* is fully compatible with both the Linux and MacOS operating systems.

In addition, *strwtrc* was designed to be user friendly and accessible, requiring minimal programming language proficiency and no prior coding experience. We intentionally kept the number of user-defined parameters to a minimum (Table 2), making it easier for users to operate the software. The default parameters were designed to work well for most cases; however, it is important to note that they may not be optimal for every possible dataset. A summary of all parameters is shown in Table 2. A comprehensive user manual and tutorial will be included with the *Situs* package to guide users through the individual stages of the approach.

## 3. Materials and Methods

Filaments in an irregular actin network, such as those found in filopodia, lamellipodia, or stress fibers, are particularly challenging to trace due to their arbitrary orientation and potential overlap or branching with other filaments. We report in the following how simulated phantom tomograms were generated from ground truth traces to provide a controlled basis for our method development and validation. Subsequently, we describe the stages of the proposed tracing framework, shown in Figure 1, to address such challenges in the tracing of actin filaments with arbitrary orientations.

### 3.1. Simulated Phantom Tomograms

In our previous work [25], we introduced *TomoSim*, a tomogram simulation approach that leverages pre-existing filament traces to generate authentic phantom tomograms, whose noise color, strength, and missing wedge were matched to experimental tomograms. In this study, we extended our simulation method to address the random orientations of the filaments.

To establish a reliable benchmark for validating *Struwwel Tracer*, we utilized actin traces of a *Dictyostelium discoideum* filopodium. A spline interpolated from the filament trace in Supplementary Data 5 from Rusu et al. [8] was volumized using a Gaussian filter with a full width at half maximum of 9 nm with a voxel spacing of 1.912 nm.

As elaborated in our previous work [25], the simulation of a tomogram aims to replicate the noise and missing wedge artifacts typically encountered in experimental maps. The simulated tomogram does not incorporate non-filamentous biological features, such as membranes. Consequently, the simulated tomogram serves as a well-defined ground truth specifically tailored to assess the accuracy of any filament-tracing framework. We used an experimental tomogram obtained from Supplementary Data 2 from Rusu et al. [8] for noise–color modeling and signal-to-noise ratio (SNR) calibration. A wedge was masked in the frequency domain to emulate a tilt range of $\pm 45^{\circ }$.

Using the color-matched noise profile, we matched the signal strength to the experimental tomogram’s SNR. We used this as the basis for testing our tracing against a range of noise levels. Noise levels were quantified as the noise intensity ratio relative to the experimental tomogram. For example, a noise level of 0.5 would have half the noise intensity of the experimental tomogram and twice the SNR. The experimental map we used for the calibration was the raw tomogram prior to any denoising or local normalization (which is typically performed on a tomogram prior to tracing [8]). Therefore, a noise level of 1.0 is a worst-case scenario that is not encountered in tracing practice. To simulate the effect of the missing denoising step, we generated a range of five equally spaced noise levels from 0.35 to 0.95, as normalized by the worst-case level. (We did not calibrate our noise on the processed tomogram [8] to avoid any subjective bias from that study and, instead, used a range of reduced noise levels as calibrated from the raw tomogram.) Note that the absolute noise scale in simulated tomograms is not strictly defined anyway because of the hidden misalignment of unknown true filament densities with the prescribed ground truth traces [25]. (So, the relative noise strengths are more relevant than the absolute scale).

### 3.2. Automatic Seed Selection

The detection of filaments in cellular tomograms is initiated from suitably chosen high-density seed points. Manual filament tracing relies on user-provided seed points, which were considered as the ground truth in previous work [6]. However, due to the labor-intensive tagging, such manual seed point selection is relatively sparse and introduces uncertainty and a subjective bias, which we wished to avoid. An automated approach is less concerned with the expense of the selection and can consider a denser oversampling of potential seed points, but it might introduce false positives. Therefore, instead of considering seed points as the definitive ground truth, we utilized them solely as starting points for the CFSs, which will be screened and refined later.

For an automatic seed point selection on a 3D grid, we identified high-density voxels within a local neighborhood. To achieve this, the tomogram was sub-partitioned into non-overlapping 3D cubes along each axis. The voxel with the highest density value within each cube was then designated as a seed voxel. In semi-regular filaments [7], the cube side length was chosen to be identical to the CFS length *l* in the CFS creation stage (below), which provides a natural length scale for the coarse-grained seed placement. However, in irregular actin networks, we expected the final seeds to be more irregularly distributed in the search cubes, so a denser sampling with a grid spacing of l/2 was implemented in *Struwwel Tracer*. For this research, we used a CFS length of l=10 voxels, resulting in seed search cubes of dimensions 5×5×5 voxels. (At a voxel spacing of 1.912 nm in the phantom tomograms above, five voxels correspond approximately to the 9 nm actin filament diameter, so the seed point density should afford a complete detection of filaments).

We assumed that seed points with local high density are necessary, but not sufficient prerequisites for true filaments. Identifying true seed voxels (that are part of a true filament) is difficult at this stage. This difficulty arises from the low SNR and the presence of missing wedge artifacts or other structures in tomograms. The determination of whether a density segment that originates in a seed belongs to a filament or not is performed in a later stage of the approach. Consequently, we refer to the short filament segments originating from a seed voxel not as predicted filament segments, but as *candidate* filament segments (CFSs).

### 3.3. CFS Creation

This stage of the approach (Figure 1) comprises two sub-steps, CFS generation and CFS refinement.

#### 3.3.1. CFS Generation

A CFS originates in the seed voxel (i,j,k) and terminates in the end voxel $\left(i^{\prime },j^{\prime },k^{\prime }\right)$, which is determined for each seed (i,j,k) by the following equations. The length *l* of the CFS in this study is user defined and is equivalent to the infinity norm of the resulting CFS vector, $l=max\{i^{\prime }-i,j^{\prime }-j,k^{\prime }-k\}$. As mentioned previously, we used a value of l=10 voxels in this paper.

On a cubic grid, let the local density at each voxel *D* be globally normalized to a range of 0 to 1. Three separate path densities, denoted as $PD_{x,y,z}$, are initialized at the seed voxel (i,j,k) for the three Cartesian *x*, *y*, and *z* axes:

(4) $$PD_{x,y,z}\left(i,j,k\right)=D\left(i,j,k\right)$$

**Figure 4:**
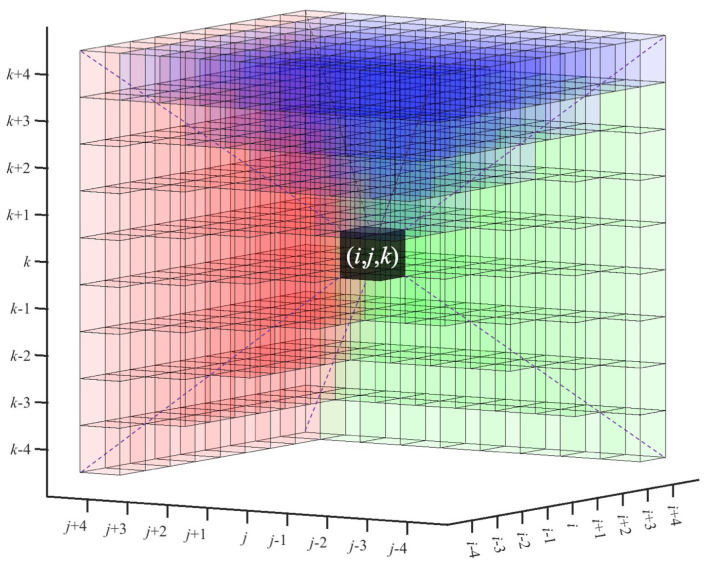
Pyramidal density search regions along different axes based on Equation (8) (green), Equation (9) (red), and Equation (10) (blue), starting from the seed voxel (black) at position (*i*,*j*,*k*). For a clearer illustration, we used a CFS length (infinity norm) l=4 voxels for the search pyramid dimensions, whereas l=10 was used elsewhere in this paper.

These path densities are then accumulated for each Cartesian axis over *l* voxels in the forward (positive) direction within the search pyramids that have the seed (i,j,k) as the vertex and can deviate by up to 45${}^{\circ }$ from the corresponding axis. The search pyramids, shown in green (*x*), red (*y*), and blue (*z*) in Figure 4, will be formally defined below. The pyramidal search approach was inspired by the *Spaghetti Tracer* algorithm [7]. However, unlike the earlier method, which was focused on a single dominant direction, we made no assumption about CFS directionality in the present work. Therefore, three Cartesian search pyramids will be combined in the following, to cover all possible orientations.

At each intermediate voxel $\left(i^{\prime \prime },j^{\prime \prime },k^{\prime \prime }\right)$ within a search pyramid, the corresponding path density (initialized in Equation (4)) is then accumulated from up to nine contributing neighbor voxels from a previous cross-section. To illustrate this, when tracing in the *y* direction (red in Figure 4), the path density $PD_{y}\left(i^{\prime \prime },j^{\prime \prime },k^{\prime \prime }\right)$ is accumulated from the preceding *y*-slice, $j^{\prime \prime }-1$ by mathematical induction. The accumulation scheme is applied to all intermediate voxels within the search pyramids:

(5) $$\begin{array}{cc} \; & PD_{y}\left(i^{\prime \prime },j^{\prime \prime },k^{\prime \prime }\right)\;=\;D\left(i^{\prime \prime },j^{\prime \prime },k^{\prime \prime }\right)\;+\\ \max_{\begin{array}{c} m,n\in \{-1,0,1\}\\ (\mathrm{if}\;\mathrm{contributing}) \end{array}} & PD_{y}\left(i^{\prime \prime }+m,j^{\prime \prime }-1,k^{\prime \prime }+n\right). \end{array}$$

Note that only neighbors m,n∈{−1,0,1} within the search pyramid are considered. Therefore, as described in [7], the accumulation zone from the seed (i,j,k) to the final endpoint $\left(i^{\prime },j^{\prime },k^{\prime }\right)$ is actually an intersection of two overlapping pyramids, i.e., path densities are accumulated only in a relatively localized region. Similarly, $PD_{x}\left(i^{\prime \prime },j^{\prime \prime },k^{\prime \prime }\right)$ and $PD_{z}\left(i^{\prime \prime },j^{\prime \prime },k^{\prime \prime }\right)$ are accumulated in their respective directions, with indices permuted accordingly:

(6) $$\begin{array}{cc} \; & PD_{x}\left(i^{\prime \prime },j^{\prime \prime },k^{\prime \prime }\right)\;=\;D\left(i^{\prime \prime },j^{\prime \prime },k^{\prime \prime }\right)\;+\\ \max_{\begin{array}{c} m,n\in \{-1,0,1\}\\ (\mathrm{if}\;\mathrm{contributing}) \end{array}} & PD_{x}\left(i^{\prime \prime }-1,j^{\prime \prime }+m,k^{\prime \prime }+n\right), \end{array}$$

(7) $$\begin{array}{cc} \; & PD_{z}\left(i^{\prime \prime },j^{\prime \prime },k^{\prime \prime }\right)\;=\;D\left(i^{\prime \prime },j^{\prime \prime },k^{\prime \prime }\right)\;+\\ \max_{\begin{array}{c} m,n\in \{-1,0,1\}\\ (\mathrm{if}\;\mathrm{contributing}) \end{array}} & PD_{z}\left(i^{\prime \prime }-m,j^{\prime \prime }+n,k^{\prime \prime }-1\right). \end{array}$$

The final forward path densities for the green (*x*), red (*y*), and blue (*z*) search pyramids in Figure 4 are then computed on the forward-facing square bases of the pyramids:

(8) $$FPD_{x}\left(i,j,k;l\right)=\max_{\begin{array}{c} j-l\leq j^{\prime }\leq j+l\\ k-l\leq k^{\prime }\leq k+l \end{array}}PD_{x}\left(i+l,j^{\prime },k^{\prime }\right),$$

(9) $$FPD_{y}\left(i,j,k;l\right)=\max_{\begin{array}{c} i-l\leq i^{\prime }\leq i+l\\ k-l\leq k^{\prime }\leq k+l \end{array}}PD_{y}\left(i^{\prime },j+l,k^{\prime }\right),$$

(10) $$FPD_{z}\left(i,j,k;l\right)=\max_{\begin{array}{c} i-l\leq i^{\prime }\leq i+l\\ j-l\leq j^{\prime }\leq j+l \end{array}}PD_{z}\left(i^{\prime },j^{\prime },k+l\right).$$

The $FPD_{\left\{x,y,z\right\}}\left(i,j,k;l\right)$ values in Equations (8)–(10) range from 0 to l+1 (number of voxels in the CFS). For the segmentation in the next stage of the algorithm, we divided the maximum between the three FPD values by l+1 to obtain a single normalized NPD value, which ranges from 0 to 1:

(11) $$NPD\left(i,j,k;l\right)=\frac{\max_{u\in \{x,y,z\}}FPD_{u}\left(i,j,k;l\right)}{l+1}.$$

The combined 3D search region (roughly a hemisphere of directions comprising the three search pyramids in Figure 4) accounts for all possible (unsigned) filament orientations. The final endpoint $\left(i^{\prime },j^{\prime },k^{\prime }\right)$ of the CFS is then defined as the point that exhibits the maximum $FPD_{u}$, according to the winning *u* axis that defines the NPD(i,j,k;l) in Equation (11).

The path-based density accumulation addresses the challenge posed by the substantial noise levels in the tomogram. Rather than solely focusing on the density of individual voxels or on density measurements along predetermined axes, the algorithm aims to detect an elongated high-density path with a specific infinity norm *l* and an orientation determined by the maximum attainable path density.

#### 3.3.2. CFS Refinement

**Figure 5:**
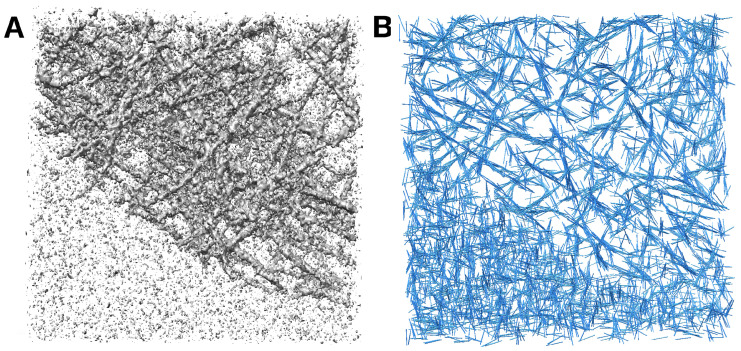
(**A**) Density map of the simulated *Dictyostelium discoideum* filopodium tomogram at a noise level of 0.50 (shown at an isocontour level of mean + 1.5 × standard deviation). Note that the cell membrane was not simulated. (**B**) Filament segments of length l=10 voxels, generated by applying the forward-path-based density accumulation and followed by backward tracing refinement.

The forward CFSs were subjected to an additional screening step based on backward tracing. From the end point $\left(i^{\prime },j^{\prime },k^{\prime }\right)$ of each forward CFS, a backward CFS with new end points $\left(i^{*},j^{*},k^{*}\right)$ was constructed by applying the above tracing algorithm in reverse. It was expected that, for a high-density CFS that is part of a true filament, the backward tracing will follow a comparable path in the reverse. Conversely, forward and backward CFS characterized by low density or noise, which are unlikely to be part of any filament, would diverge from each other. To determine the similarity of paths between forward and backward tracing, the angle between the points (i,j,k), $\left(i^{\prime },j^{\prime },k^{\prime }\right)$, and $\left(i^{*},j^{*},k^{*}\right)$ was calculated, and a fixed angle threshold of 20${}^{\circ }$ was employed to screen out inconsistent forward and backward CFSs. An example of the intermediate results after the refinement stage is shown in Figure 5. The surviving CFSs (Figure 5B) exhibited the preferred orientation of filaments in the actin-rich interior of the cell, whereas the exterior of the cell (lower left) yielded unstructured CFS patterns, which were not true filaments and will need to be filtered out in the following.

### 3.4. CFS Segmentation

Figure 5B illustrates the segmentation problem in the irregular actin networks, which are the focus of this work. In earlier work on ordered actin filament bundles (with a dominant orientation), we found that it was possible to screen out spurious CFSs (such as in the lower left corner) using an automated binning method that finds a suitable NPD threshold above which the CFSs remain contained in the true filament region. CFSs with NPD values below this threshold were then eliminated automatically. We propose here to use a similar NPD threshold segmentation. However, irregular actin network CFSs exhibit low NPD contrast (there was no separate directional denoising stage as in regular filaments [7]), and we did not assume that irregular networks are localized in a specific region. Therefore, it would be desirable to fine-tune the threshold as a continuous parameter instead of prescribing discrete bin values. We implemented a novel way to allow users to perform such a continuous thresholding quickly and conveniently with a third-party molecular graphics program such as VMD [26], Sculptor [8] or UCSF Chimera [22]. Our approach outputs a so-called “pruning map”, which contains the NPD values masked by the CFS locations. Voxels in close proximity (i.e., one voxel) to the CFSs are assigned the NPD value (Equation (11)) of the CFS. When a voxel can draw NPD values from multiple CFSs, the highest value is retained.

**Figure 6:**
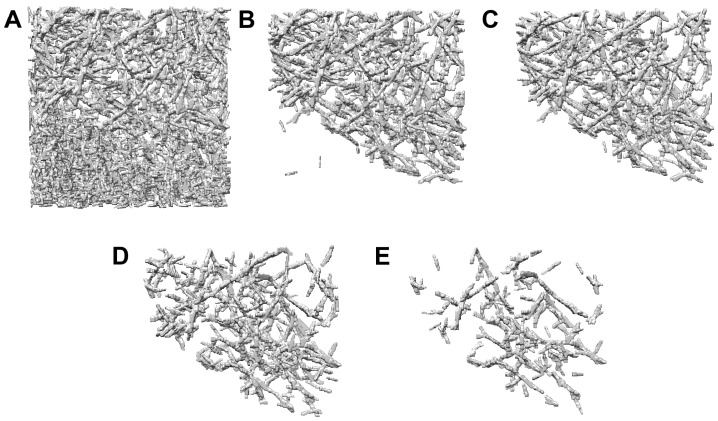
Pruning map of the simulated tomogram in Figure 5A (noise level: 0.50) at different isocontour levels: (**A**) 0.40, (**B**) 0.45, (**C**) 0.50, (**D**) 0.55, and (**E**) 0.60.

In Figure 6, the pruning map returned by *Struwwel Tracer* is shown at different isocontour levels. By adjusting the level in the histogram window, users can visually determine the threshold in the pruning map (i.e., the threshold NPD) that represents likely filament segments. An overly low threshold would result in numerous false positives (Figure 6A), whereas an overly high threshold would lead to a significant number of false negatives (Figure 6E). Visual examination indicates that the isocontour values within the range depicted in Figure 6C,D are likely to represent the filaments accurately. Note that the subsequent steps of the algorithm are not very sensitive to the selected threshold value because of the screening and gap filling that still follow. The main purpose of the visual segmentation was to suppress spurious filaments, as is shown in Figure 6A, so a reasonable guess of the threshold parameter suffices in practice.

### 3.5. CFS Fusion

In the filament-fusion stage, we employed multiple geometric strategies to combine short, linear CFSs (Figure 5B) into curved, longer filaments. To account for the irregular organization of actin networks, the following fusion steps were specifically designed for *Struwwel Tracer* and were different from the collinearity test we developed earlier for oriented filaments [7].

#### 3.5.1. Fusion Based on Physical Proximity

The initial fusion step considers the relative spacing and orientation of neighboring CFSs. Adjacent filaments that exhibit similar orientations (default angle tolerance of CFS center lines: 30${}^{\circ }$) will be connected. We determined if two adjacent CFSs overlap or touch each other based on their spacing along the center lines of the CFSs (the default gap tolerance was 10 voxels). CFSs that meet the criteria are combined by connecting the end point of a CFS to the starting point of the successive CFS. Since it is not required that two CFSs exhibit perfectly matched orientations and positions before they are merged, we smoothed the center line of the fused CFSs by sampling, interpolation, and the removal of redundant points.

#### 3.5.2. Fusion by Extension

In addition to the proximity fusion of short CFSs, we tested whether a one-time extension of CFSs by a length *l* can extrapolate CFSs such that they make contact with another CFS using the same angle and spacing criteria. The extension step aimed to fill up any noise-induced gaps present in the filaments. Due to the high noise levels and missing wedge artifacts in the tomograms, it is possible that true filaments exhibit regions of density at the level of the noise, which may be excluded by a high threshold during the visual inspection of the pruning map (Figure 6). We, therefore, examined whether the one-time extension by a length *l* can help to fuse more CFSs. Similar to the previous step, we smoothed the center lines of fused CFSs by sampling, interpolation, and the removal of redundant points.

## 4. Conclusions

The release of *Struwwel Tracer* is the capstone of several years of development effort on actin filament tracing that extends and completes our set of efficient solutions for hexagonal, closely packed bundles (*BundleTrac* [6]) and semi-regular bundles with a dominant direction (*Spaghetti Tracer* [7]) to irregular networks of randomly oriented actin filaments. *Struwwel Tracer* also completes the paradigm shift we began with *Spaghetti Tracer*, when we first used a dynamic-programming-based method at the voxel level that does not require an expensive missing wedge correction, template convolution, or deconvolution. Therefore, both *Spaghetti Tracer* and *Struwwel Tracer* yield a substantial improvement in time efficiency over earlier template convolution or deconvolution approaches. This development was guided by a rigorous optimization of the performance using a statistical F1-score analysis enabled by simulated phantom tomograms.

The proposed framework incorporates a directional path search in all three Cartesian directions (*x*, *y*, and *z*) to ensure a comprehensive coverage of filament orientations. The algorithm identifies the path with the highest density, generating short filament segments, which are subsequently combined to form the final filament traces. The evaluation using F1-scores on simulated tomograms proved the algorithm’s high efficacy in filament tracing. Visual inspection of the results further confirmed its agreement with experimental tomograms. Our approach is robust and fast, works with simple parameter settings, and can deliver a comprehensive performance on simulated and experimental datasets. Moreover, in contrast to deep-learning-based approaches that rely on substantial processing power, such as dedicated GPUs, the implementation in the *Situs* package does not necessitate such resources. It can seamlessly operate on any conventional laptop or desktop computer with a standard CPU.

In addition to the high efficacy in tracing, the proposed framework is also robust as it is, in principle, capable of tracing various types of filaments, whether they are randomly oriented networks or regular (with a dominant direction). We note, however, that our earlier tools (*Volume Tracer* [8], *BundleTrac* [6], *ConDe* [4], and *Spaghetti Tracer* [7]) have unique features that were optimized for their specific applications (e.g., an additional capability to detect alpha helices in *Volume Tracer*, the use of hexagonal bundle templates in *BundleTrac*, the ability to prescribe diverse shape templates in *ConDe*, and an integrated directional denoising that takes advantage of an ordered filament arrangement in *Spaghetti Tracer*). A comprehensive F1-score comparison of various tools and conditions could be performed in a future review.

As the study of the actin cytoskeleton is of increasing importance in biology, the proposed tool will become useful to structural biologists in need of free, open-source software solutions. Although this manuscript focused primarily on the computational aspects of actin filament tracing, the developed framework can be applied by any experimental lab to newly acquired experimental tomograms of the actin cytoskeleton. Functionality for measuring actin filament length distributions and the angles between filaments, and for labeling branch junctions [10,23], can be included in future work.

## Figures and Tables

**Table 1:** A performance comparison of the proposed *Struwwel Tracer* approach for simulated tomograms of a *Dictyostelium discoideum* filopodium (see Section 3.1) at various noise levels.

Noise Level	Precision	Recall	F1-Score
0.35	0.97	0.85	0.90
0.50	0.97	0.81	0.88
0.65	0.96	0.84	0.89
0.80	0.95	0.79	0.87
0.95	0.95	0.78	0.85

**Table 2:** Command line parameters of the *strwtrc* program implemented in *Situs* [8] and corresponding stages of the approach (see Section 3.2, Section 3.3, Section 3.4 and Section 3.5 and the Data Availability Statement).

Parameter Name	*strwtrc* Argument	Description	Default Value	Program Stages
**Required Parameter**				
Threshold	-thr	Threshold for partitioning the CFS by the normalized path density.	N/A (user-defined based on the pruning map)	CFS segmentation
**Optional Parameters**				
Length	-len	Length (infinity norm) of the CFS in voxel units. Internally, this also defines the spacing of the cubic grid for placing seed points (half this value; see the text) and the extension length of the CFS (same value).	10	Automatic seed selection, CFS generation, and CFS fusion
Gap Spacing	-gap	Maximum gap spacing, in voxels, tolerated while fusing adjacent CFSs.	10	CFS fusion
Fusion Angle	-ang	Maximum angle, in degrees, tolerated while fusing adjacent CFSs.	30	CFS fusion

## Data Availability

The source of *Struwwel Tracer* can be freely downloaded at https://situs.biomachina.org in *Situs* version 3.2.
